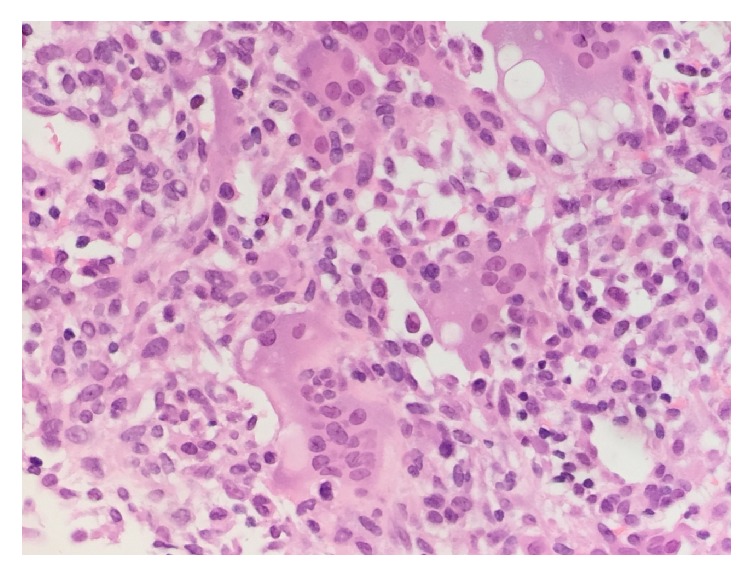# Denosumab Therapy for Giant Cell Tumor of Bone Pulmonary Metastasis

**DOI:** 10.1155/2017/2302597

**Published:** 2017-05-10

**Authors:** Ryan Carlisle Egbert, Ryan Folsom, Jeff Bell, Rajiv Rajani

**Affiliations:** ^1^Department of Orthopaedics, University of Texas Health San Antonio, MC 7774, 7703 Floyd Curl Drive, San Antonio, TX 78229-3900, USA; ^2^University of Texas School of Medicine at San Antonio, 7703 Floyd Curl Drive, San Antonio, TX 78229-3900, USA; ^3^Department of Anesthesiology, Kansas University School of Medicine-Wichita, 929 N. St. Francis, Room 8074, Wichita, KS 67214, USA; ^4^Orthopaedic Oncology, Department of Orthopaedics, University of Texas Health San Antonio, MC 7774, 7703 Floyd Curl Drive, San Antonio, TX 78229-3900, USA

## Abstract

**Case:**

A 68-year-old female was diagnosed with giant cell tumor of bone (GCTB) metastatic to her lungs. The patient was treated with IV denosumab for the course of 4.5 years for these metastases. The metastatic tumor burden decreased significantly after only 3 months of therapy. The size of the metastases has been stable for over 4 years.

**Conclusion:**

Denosumab therapy has promise in the treatment of GCTB, including pulmonary metastasis. However, the long-term role of denosumab for pulmonary metastases is yet to be determined.

## 1. Introduction

Giant cell tumor of bone (GCTB) is a benign yet aggressive lytic tumor of bone occurring in the metaphysis and epiphysis of long bones. The most common locations involved are the distal femur, proximal tibia, and distal radius [1]. Such destructive lesions were first described by Cooper and Travers [2] in 1818, but GCTB was not distinguished from other tumors of bone until 1940 [3]. GCTB typically presents in persons 20 to 40 years old and accounts for approximately 20% of all benign tumors of bone [4, 5]. Local recurrence was first described by Virchow [4] and occurs at a rather high rate (18%–50%) [6]. Metastasis occurs rarely, typically in the setting of locally recurrent GCTB. The most common site of metastasis is to the lungs [7]. Metastases are considered benign, are histologically identical to the primary tumor [8], and rarely contribute to death in the majority of patients [7].

The mainstay of treatment of GCTB pulmonary metastasis is surgical [9]. Since pulmonary metastasis of GCTB is rare, there are still no studies comparing different treatment modalities in the current literature. Faisham et al. proposed that aggressive treatment with surgical excision of lung metastasis is mandatory for aggressive GCTB. This is now widely accepted as treatment of choice [10]. Additionally, a case-control study done by Tse et al. suggested medical management as a promising avenue for GCTB treatment [11].

The histologic composition of GCTB consists of sheets of neoplastic spindle shaped mononuclear cells with high levels of RANK Ligand (RANKL) expression, intermixed with large osteoclast giant cells [8, 12]. RANKL potentiates the osteoclast differentiation of monocytes leading to the lytic nature of the tumor [12]. Denosumab is a human monoclonal antibody that inhibits normal and tumor associated RANKL [13–15]. Some studies have shown that denosumab has the ability to prevent tumor progression, induce primary tumor reduction, increase bone formation, and reduce pain in patients with GCTB [16–18]. However, research has not yet shown a definitive role for denosumab in managing pulmonary metastasis of GCTB.

## 2. Case Report

**Figure 1:**
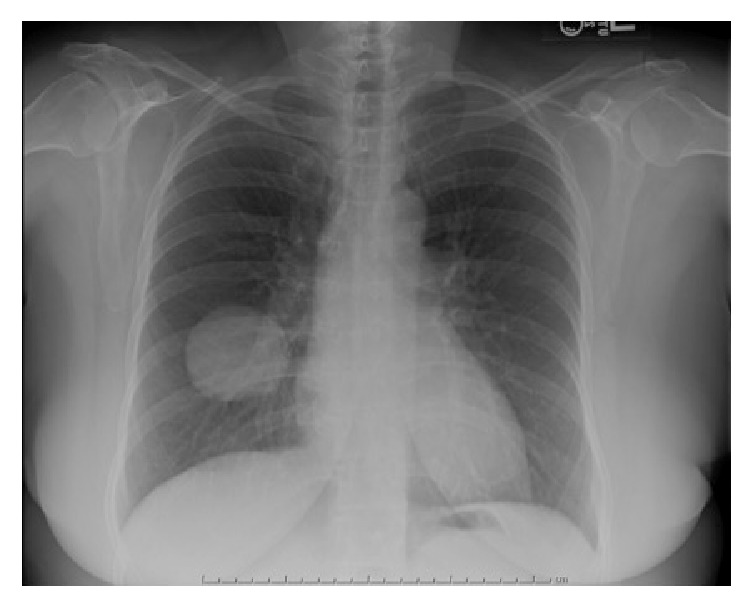


**Figure 2:**
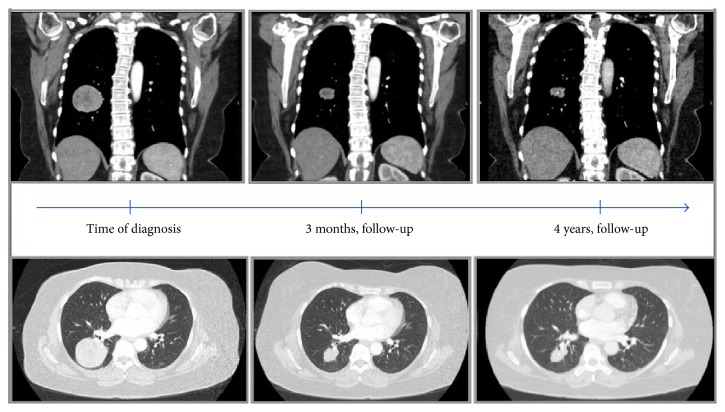


Our case is a 68-year-old female with a past medical history of hyperlipidemia and insulin-controlled type 2 diabetes mellitus who presented with right knee pain and swelling. She denied any history of trauma. Radiograph and magnetic resonance imaging were concerning for GCTB. She underwent curettage and cementation of the right distal femur lesion and histological examination confirmed the suspected diagnosis of GCTB. Approximately one year later, she developed local recurrence of the tumor, which was also curetted with additional use of cement. One year after the second surgery, a routine chest radiograph showed lung lesions suspicious of metastatic disease (Figure 1). At the time she was feeling well, with only mild pain in her right knee. Computed tomography (CT) imaging verified the presence of several pulmonary nodules including a large right lower lobe (RLL) mass measuring 5.6 cm (Figure 2). CT-guided core needle biopsy of the largest lung mass confirmed metastatic GCTB (Figure 3).

After discussion at a tertiary center musculoskeletal tumor board the patient was started on denosumab therapy. Treatment was initiated with a loading dose of 120 mg every week for three weeks. She was then transitioned to receive a dose of 120 mg every four weeks. She was also placed on daily calcium and vitamin D supplements.

At 3 months after the initiation of treatment she had repeat CT imaging of her chest. The scan showed the RLL mass had decreased considerably in size, now measuring 2.6 cm in its greatest dimension. There was also a decrease in size of the other pulmonary lesions (Figure 2). No new pulmonary nodules were seen. Since then she has had CT imaging of her chest every three months and radiographs of her right knee every six months. She has been without subjective complaint at each follow-up visit. After 16 months of denosumab therapy, q cardiothoracic surgery consultation was obtained and, due to the location and stability of disease, no surgery was recommended. The patient also was adamantly opposed to surgery. She continued to tolerate the denosumab without any evidence of side effects or complications.

Forty-eight months after initiating treatment, CT imaging showed the stable RLL mass as well as the smaller pulmonary lesions to be unchanged in size (Figure 2).

## 3. Discussion

We present a case of metastatic GCTB successfully treated with denosumab for 4 years. A literature search was able to find only one similar case. This was in a 17-year-old female who was followed for only three months while on denosumab [19]. Our patient has been followed now for over 4 years without signs of disease progression.

Dosing of the denosumab was also different in the two cases. In our patient, we followed the treatment regimen first described by Thomas et al. which consists of three loading doses of 120 mg seven days apart followed by one dose every four weeks [20]. In the case of the 17-year-old female, Demirsoy et al. used a 120 mg monthly dose without loading while achieving similar results of tumor regression [19]. Further studies are required to determine the optimum dose.

A denosumab safety study conducted by Chawla et al. showed low rates of hypocalcemia, osteonecrosis, neutropenia, hypophosphataemia, anemia, back pain, and pain in the extremities [20, 21]. Patients were followed for up to one year. Because of these findings, we supplemented our patient with prophylactic vitamin D and calcium. Our patient has yet to complain of bone pain, back pain, or other symptoms potentially attributable to denosumab therapy. While this treatment may be promising, broader long-term follow-up of patients treated with denosumab would be beneficial.

While denosumab has proven to be effective in reducing metastatic tumor size through interfering with osteoclastic differentiation, it does not target the neoplastic stromal cells, which will continue to proliferate with discontinuation of therapy. It is unknown if patients require chronic denosumab therapy or if the rate of neoplastic stromal cell proliferation can be reduced sufficiently with initial treatment that patients can stop therapy without progression of disease [22]. Recent studies suggest that discontinuation of therapy would lead to regrowth of tumor.

Additionally, there is a theoretical increased risk of pathologic fracture with chronic use of medications similar to bisphosphonates. Therefore, we had considered discontinuing denosumab after three years of therapy. However, due to a report in the literature of critical hypercalcemia following cessation of denosumab [23], the lack of consensus on long-term denosumab therapy [24], and our patient's lack of adverse effects while on the medication we decided to continue treatment. Also, with our patient refusing surgical excision as a form of treatment, the only options that remain are continuation or cessation of denosumab therapy. At this time, no plans for discontinuation exist.

Other questions concerning denosumab therapy remain. More studies are needed to determine its application in all the stages of GCTB. We are hopeful denosumab can be a reasonable alternative to surgical treatment for pulmonary metastasis [22]. The frequency of dosing and duration of denosumab treatment have yet to be established as well [25]. While denosumab appears effective, further studies are needed to test its superiority over cheaper and more readily available medications [21].

## 4. Conclusion

Denosumab therapy has promise in the treatment of GCTB, including pulmonary metastasis. However, there is still much to do in defining its use in treating the different stages of GCTB. Also needed are long-term studies further defining its optimum dose, side effects, and how the disease progresses after discontinuation.

## Figures and Tables

**Figure 3:**